# Rapid changes in seed dispersal traits may modify plant responses to global change

**DOI:** 10.1093/aobpla/plz020

**Published:** 2019-03-28

**Authors:** Jeremy S Johnson, Robert Stephen Cantrell, Chris Cosner, Florian Hartig, Alan Hastings, Haldre S Rogers, Eugene W Schupp, Katriona Shea, Brittany J Teller, Xiao Yu, Damaris Zurell, Gesine Pufal

**Affiliations:** 1 School of Forestry, Northern Arizona University, Flagstaff, AZ, USA; 2 Dorena Genetic Resource Center, USDA Forest Service, Cottage Grove, OR, USA; 3 Department of Mathematics, The University of Miami, Coral Gables, FL, USA; 4 Theoretical Ecology, University of Regensburg, Regensburg, Germany; 5 Department of Environmental Science and Policy, University of California, Davis, CA, USA; 6 Department of Ecology, Evolution, and Behavior, Iowa State University, Ames, IA, USA; 7 Department of Wildland Resources & Ecology Center, Utah State University, Logan, UT, USA; 8 Department of Biology, The Pennsylvania State University, University Park, PA, USA; 9 Department of Geography, Humboldt-University Berlin, Berlin, Germany; 10 Department of Land Change and Science, Swiss Federal Institute WSL, Birmensdorf, Switzerland; 11 Nature Conservation and Landscape Ecology, University of Freiburg, Freiburg, Germany

## Abstract

When climatic or environmental conditions change, plant populations must either adapt to these new conditions, or track their niche via seed dispersal. Adaptation of plants to different abiotic environments has mostly been discussed with respect to physiological and demographic parameters that allow local persistence. However, rapid modifications in response to changing environmental conditions can also affect seed dispersal, both via plant traits and via their dispersal agents. Studying such changes empirically is challenging, due to the high variability in dispersal success, resulting from environmental heterogeneity, and substantial phenotypic variability of dispersal-related traits of seeds and their dispersers. The exact mechanisms that drive rapid changes are often not well understood, but the ecological implications of these processes are essential determinants of dispersal success, and deserve more attention from ecologists, especially in the context of adaptation to global change. We outline the evidence for rapid changes in seed dispersal traits by discussing variability due to plasticity or genetics broadly, and describe the specific traits and biological systems in which variability in dispersal is being studied, before discussing some of the potential underlying mechanisms. We then address future research needs and propose a simulation model that incorporates phenotypic plasticity in seed dispersal. We close with a call to action and encourage ecologists and biologist to embrace the challenge of better understanding rapid changes in seed dispersal and their consequences for the reaction of plant populations to global change.

## Teaser

Global ecological change is causing plant populations to either adapt or move in response to new environmental conditions. In many species it is thought that seed dispersal may not allow populations to move at a rate that tracks the shifting climate. However, phenotypic plasticity and rapid genetic changes in traits associated with seed dispersal may modify the response capabilities of plants and allow them to responds more quickly. We explore the evidence for rapid modification of seed dispersal traits and propose a path forward to better understand the ways in which seed dispersal may buffer the effects of global ecological change.

## Introduction

Global environmental change, in all its forms, is considered one of society’s greatest challenges today. When climatic envelopes shift (Loarie *et al.* 2009; Mahony *et al.* 2017), or when frugivore communities change due to invasion or over-exploitation (Bleher and Böhning-Gaese 2001), plant populations can react by either adapting locally to the new environmental conditions (Cochrane *et al.* 2015) or by dispersing, attempting to track their climatic envelope or ecological niche (Corlett and Westcott 2013; Johnson *et al.* 2016; Graae *et al.* 2018). If their dispersal abilities do not match the shift of their ecological niche, a rapid change in, or dependence on, dispersal ability could allow them to respond. Whilst rapid changes in plant traits, such as phenology, mortality and metabolic processes to changed environmental or biotic conditions have been addressed relatively widely (Nicotra *et al.* 2010; Bardgett *et al.* 2014; Gratani 2014; Graae *et al.* 2018), rapid changes in traits affecting plant movement have received considerably less attention, and few studies report evolutionary responses on these traits.

A possible interpretation of this lack of empirical evidence for adaptation of seed dispersal is that selection in plants acts on the immediate factors regulating local survival rather than a process that determines the location of seeds, which then influences survival. However, given the importance of dispersal for the inclusive fitness of a plant, we contend that adaptive changes in seed dispersal do occur. The more plausible explanation for the lack of evidence is that plant movement (dispersal) is notoriously difficult to study (Rogers *et al.*, this issue-a). We hardly understand dispersal as it is, making it challenging to detect changes in dispersal processes due to global change. Moreover, one might conjecture that dispersal becomes more important as climate change progresses and increasing mismatches between current plant distributions and their environmental optima intensify.

For dispersal traits to change rapidly in response to changes in the biotic and abiotic environment, phenotypic diversity associated with dispersal must be present or phenotypes must respond in a plastic manner when they encounter novel environments. Dispersal, defined as the movement of a propagule from its natal source with consequences for gene flow through space (Ronce 2007), has a complex, mostly polygenic, genetic architecture. The speed at which dispersal traits, or suites of traits, can change is associated with the underlying genetic sequence, phenotypic variation in dispersal traits and their covariance with other traits under selection (Saastamoinen *et al.* 2018), and/or epigenetic mechanisms leading to plastic responses to environmental variability. Whether the mechanisms of rapid changes of dispersal traits are the product of genetic or plastic variation, the outcomes are similar because improved dispersal ability will similarly increase population spread potential.

**Figure 1.**
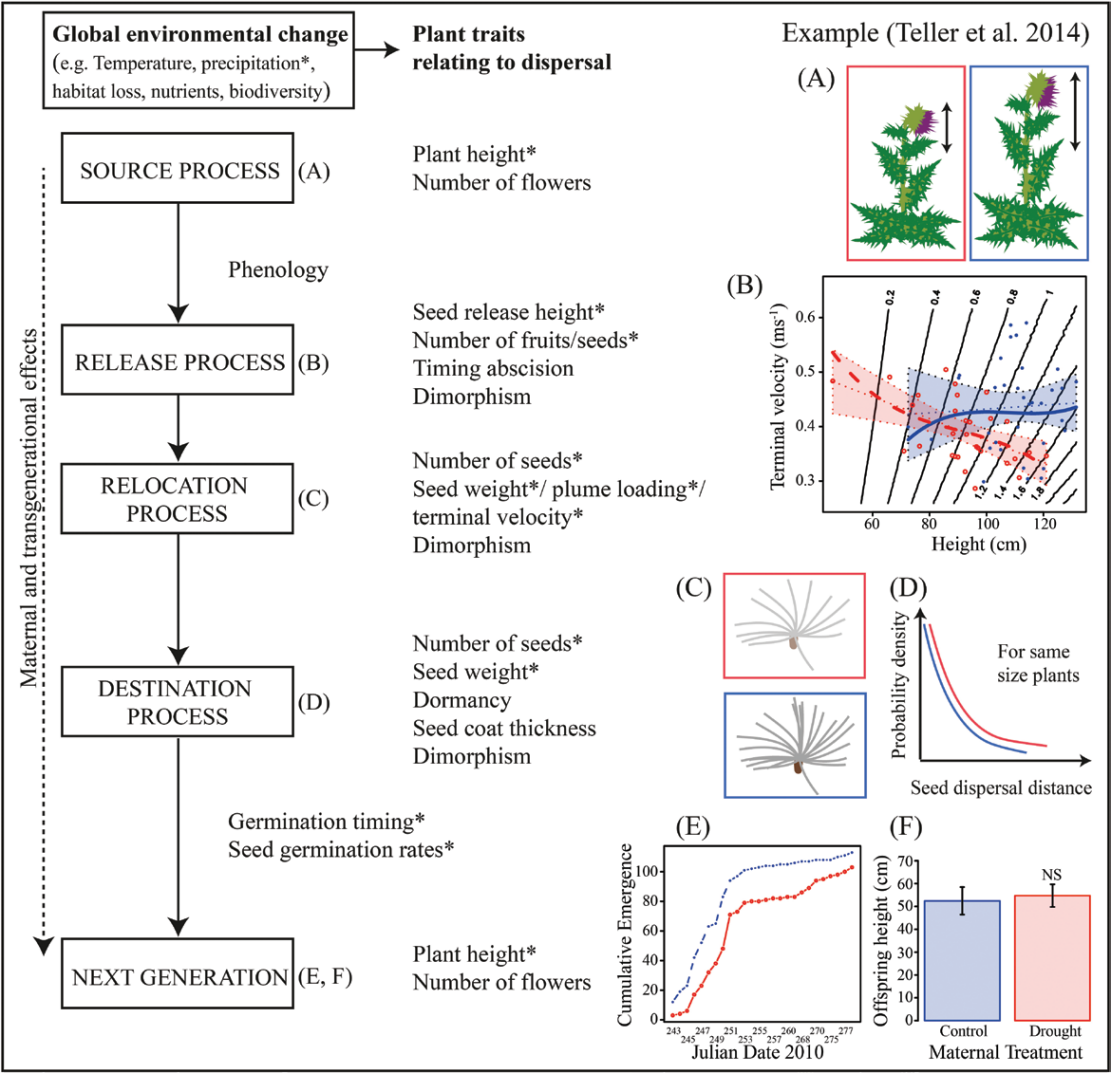
Dispersal stages, and possibilities of their modification via rapid responses of plant dispersal traits to global environmental change. Global environmental change includes changes in temperature, precipitation, habitat availability, biodiversity and nutrient availability, which in turn can elicit rapid responses in plant traits affecting their dispersal. These responses may affect traits of the maternal plant up to seed release (Source processes), traits that directly influence the initiation of dispersal (maternal or seed traits) (Release processes) and the dispersal process itself (seed traits) (Relocation process), traits that are important after the dispersal process (seed traits) (Destination process) and might even affect the performance of the next generation (maternal and transgenerational effects) (Process stages Jongejans *et al.* 2015). Potential consequences of a rapid response of dispersal traits to climate change are exemplified with a study by Teller *et al.* (2014). They studied the effect of drought on varying dispersal traits during different stages of the dispersal process (traits with *) in *Carduus nutans*. Drought conditions (red figures) reduced plant and seed release height, whereas well-watered plants (blue figures) showed more variability in their height (A). Interestingly, seeds from taller *C. nutans* plants in the drought treatment should disperse at least as far as same-sized individuals under well-watered conditions due to a decrease in seed terminal velocity in the drought treatment (B). Under drought stress, phenotypic plasticity of maternal plant and seed traits (C) could hence favour longer distance dispersal (D) but with the cost of fewer seeds that might also germinate later (E). Maternal effects for the next generation could not be observed in this example (F). Panels (B), (E) and (F) are reproduced with permission from Teller *et al.* (2014).

Our goal in this perspective is to call attention to current research on the potential of rapid changes in seed dispersal as a response to global change. We highlight that, despite some progress, our understanding of the variability regulating seed dispersal remains poor. This is primarily because of the high intrinsic variability of dispersal-related traits of seeds and their dispersers coupled with the high variability in effective dispersal resulting from environmental heterogeneity. Moreover, the logistical challenges, such as long lifespan and the large number of seeds produced, make studying rapid changes in seed dispersal difficult, and few studies to date have tackled these questions. We organize our essay by first discussing the specific traits and biological systems in which changes in dispersal are being studied, along with the ways in which these questions are currently being addressed. Then we discuss potential mechanisms that could result in rapid changes in seed dispersal. We close with recommendations of future research needs and propose a model that incorporates phenotypic plasticity while stressing the need for more empirical studies to untangle the role of genetic and plastic contributions to adaptation of seed dispersal. We show that there is evidence for rapid changes in seed dispersal through multiple mechanisms and that they may allow plants to respond quickly to changing environments (Fig. 1).

## Traits Important to Seed Dispersal

Several traits are important contributors to patterns of seed dispersal. These traits or suite of traits are variable at the subindividual level in their range of phenotypes and plant fitness can be both influenced and mediated by environmental variation (Schupp, this issue; Snell et al. 2019) through different pathways. Though specific traits vary depending on dispersal mode (e.g. anemochory vs. epizoochory) and life-history strategies (Beckman *et al.* 2018), here we have grouped them into three primary types: traits related to movement, phenology and longevity. Plasticity of movement-related traits—e.g. those associated with attractiveness to frugivores, attachment to dispersers or aerodynamics—is the most obvious, but plants may also vary in phenology/timing of propagule production, or in the length of dormancy on the plant or the ground, with consequences for seed dispersal and hence impacts on plant fitness.

Traits that influence abiotic seed dispersal are largely related to the structure of the seed or plant (see Fig. 1). For wind-dispersed species, seed weight affects falling velocity (Teller *et al.* 2014), plant height affects distance (Thomson *et al.* 2011; Zhang *et al.* 2012) and seed morphology influences the dispersal kernel (Augspurger and Franson 1987). In *Arabidopsis thaliana*, plant density influences dispersal distance (Wender *et al.* 2005). Both phenotypic plasticity and genetic variation are responsible for reduced dispersal at the range limits of *Cakile edentula* (LaRue *et al.* 2018). For epizoochorous species, the type of attachment affects the likelihood of hitching a ride on a passing animal. Some wind-dispersed and epizoochorous species have heteromorphic seeds (e.g. Martorell and López 2014) which allows them to utilize multiple dispersal vectors, and rapid evolution of a second seed type with a different dispersal mode has been shown to occur within 5–12 generations of selection (Cheptou *et al.* 2008).

In endozoochorous systems, a complex suite of traits may influence movement, as plants typically respond to a dynamic community of frugivores and may use a variety of traits to attract frugivores. These include pulp:seed ratio, colour, volatiles, fruit size, fruit shape, seed size and shape. There is evidence that fruits respond to the environment in a plastic manner. For example, nutritional quality changed with temperature in cherry tomato (Gautier *et al.* 2005), fruit and seed size, nutritional content, and pulp:seed ratio responded to soil water availability and nutrients in a desert shrub (Lotan and Izhaki 2013). Fruit weight and seed number were related to rainfall and temperature in a small Mediterranean tree (Chiarucci *et al.* 1993). In a study of intraspecific variation of 63 fleshy-fruited plant species, fruit size was associated with rainfall, and carbohydrate and lipid content decreased with latitude (Hampe 2003). While there is evidence for plasticity in fruit and seed traits in response to their abiotic environment, there are limited examples of rapid changes in fruit traits to changing biotic environments except for one study finding rapid change in seed size in response to a shifting frugivore community (Galetti *et al.* 2013). However, rapid change in fruit size, through artificial selection, is a key component of domestication of agricultural crops, and therefore is likely more common than it appears in the literature. For example, in tomatoes, a single loci, *fw2.2*, has a large impact on fruit size and was important during domestication (Frary *et al.* 2000).

Rather than changing physical traits, plants may adjust the timing of propagule production or ripening in response to changing environments. For example, variation in temperature and precipitation has been show to alter flowering time (Jordan *et al.* 2015) and some epigenetically regulated (discussed below) hypomethylated regions have been associated with earlier flowering (Fieldes *et al.* 2005) or seed production (Yakovlev *et al.* 2012). The timing of fruit/seed production and abscission in plants may respond to environmental factors as well. Ethylene plays a key role in the timing of ripening for many agricultural species (Barry and Giovannoni 2007), and may be related to the timing of ripening and abscission in wild species closely related to tomato (Grumet *et al.* 1981), yet the role of ethylene is largely unexplored in wild plants. In Costa Rica, the ripening rate of fruit was linked to frugivore visitation rate, such that fruit ripened more rapidly when frugivore visitation was higher (Levey 1987).

Finally, plants may respond to a changing biotic or abiotic environment by relying less on dispersal through space and more on dispersal through time. Traits related to seed longevity in the canopy or soil seed bank may allow plants to persist longer in situations of reduced dispersal. Traits such as seed hardness, physical protection, chemical protection, flesh quantity and likelihood of microbial attack tend to trade-off with traits related to movement (e.g. attractiveness to dispersers). For example, the annual grass *Aegilops triuncialis* has dimorphic seeds and late season precipitation induces extended seed dormancy in one of the seed types (Dyer 2017).

## Rapid Changes in Dispersal in Different Systems

To date, changes in dispersal (or more broadly movement) have been studied in both terrestrial and aquatic systems, though few studies have addressed rapid changes. We specifically highlight responses to effects of global environmental change (Table 1). Most of the studies on dispersal have addressed phenotypic variation across a range of external conditions empirically without investigating the degree to which the phenotypic trait variation stems from genetic or epigenetic factors. In light of rapid genetic responses to environmental changes in other traits (Hoffmann and Sgrò 2011), the heritability of various dispersal traits and their genetic architecture should be of high importance, but heritability of dispersal traits is scarcely known outside of a few traits in annual crops and model plant systems (see the review by Saastamoinen *et al.* 2018 and citations therein).

**Table 1.** Environmental conditions affected by global environmental change, which have been shown to elicit a rapid response in dispersal ability. Given are examples of studies, with conclusions drawn about the main consequence for the dispersal ability of the respective study organisms.

Global environmental threat	Variables	Exemplary studies	Main consequence for dispersal ability
Plants			
Climate change	Temperature increase	Zhang *et al.* (2012)	Increase in dispersal distance
		Teller *et al.* (2016)	Increase in probability of seed release
		Li *et al.* (2018)	Increase in fruit dehiscence—phenology of dispersal
	Drought	Teller *et al.* (2014)	Increase in dispersal distance
	Decrease in water availability	Martorell and López (2014)	Increase in highly dispersible seeds
Habitat fragmentation	Increase in distance of suitable habitat patches	Cheptou *et al.* (2008)	Increase of proportion of non-dispersing seeds in highly fragmented habitat
Nutrient cycling	Nutrient depletion	Imbert and Ronce (2001)	Increase in seeds with dispersal structures
Biodiversity loss	Loss of dispersal vectors	Galetti *et al.* (2013)	Decrease in seed size—negative effects on population fitness
Animals			
Climate change	Temperature increase		
	Drought	Rozen-Rechels *et al.* (2018)	Increased anxiety (maternal effects) and greater exploration behaviour
	Precipitation, extreme weather events	Loe *et al.* (2016)	Behavioral plasticity leads to increased movement to favourable habitats
	Ocean acidification	Rossi *et al.* (2018)	Maladaption to environmental cues to unsuitable habitat in larval fish
	Winter weather conditions	Gurarie *et al.* (2017)	
	Wind	Vansteelant *et al.* (2017)	
Habitat fragmentation		Moraes *et al.* (2018)	
	Comparison extant metapopulation with fragmented extinct population	Fountain *et al.* (2016)	Selection for genotypes with higher colonization capacity in fragmented landscapes in butterflies

To illustrate some of the population and community level impacts that changes in dispersal traits can have, we discuss changes in plant traits, changes in external conditions and changes in animal disperser traits. Including animal dispersers not only gives a more complete picture of the systems in which rapid responses in dispersal are addressed (even if the mechanisms may be different), but also demonstrates how seed dispersal can be affected when biotic dispersal vectors respond to environmental changes and thus influence the total dispersal kernel (Rogers *et al.*, this issue-b). To be comparable with plant dispersal, we consider animal movement broadly, including such topics as natal dispersal, seasonal migration, foraging movement, settlement behaviour and movements within home ranges.

### Change in plant traits

Changes in seed dispersal have been mostly studied experimentally within the context of phenotypic variability of dispersal traits (Fig. 1). A common approach has focused on environmental influences on heteromorphic species that produce two or more distinct seed morphs that differ in dispersal potential. Environmental influences on the proportion of more dispersible morphs include herbivory (Fukano *et al.* 2014) or environmental stress (Mandák and Pyšek 1999; Imbert and Ronce 2001; Cheptou *et al.* 2008; Martorell and López 2014). Other studies have considered environmental influences on continuous variation in dispersal traits, again, addressing the effects of herbivory (de la Pena and Bonte 2014; Marchetto *et al.* 2014) and environmental stress (Zhang *et al.* 2012; Teller *et al.* 2014) on traits related to wind dispersal such as pappus length, plume loading and plant height. Moreover, the timing of seed release can change in response to the growth conditions experienced by maternal plants (Teller *et al.* 2016; Li *et al.* 2018). Similarly, effects of water and soil nutrients on traits associated with endozoochorous seed dispersal such as fruit and seed size, pulp-to-seed ratio and pulp nutrients have been experimentally investigated (Lotan and Izhaki 2013).

Less work has explicitly addressed rapid changes in seed dispersal, although studies have considered within- and among-population genetic variation in traits associated with dispersal (see Saastamoinen *et al.* 2018 for a review of genetics in seed dispersal) which demonstrate the long-term potential for evolution of dispersal (e.g. Riba *et al.* 2005). Perhaps the most elegant demonstration of rapid change in dispersal is the reduction in *Euterpe edulis* fruit size and increased dispersal distance, associated with the selective defaunation of large dispersers in the Atlantic forest of Brazil (Galetti *et al.* 2013; Carvalho *et al.* 2016). Studies have also demonstrated directional selection by seed dispersers on a variety of fruit display traits relevant to seed dispersal such as fruit sugar concentration (Palacio *et al.* 2014), fruit/seed size (e.g. Wheelwright 1993; Hernández 2008) and ripening phenology (e.g. Alcantara *et al.* 1997); however, these studies have not demonstrated an evolutionary response (but see Heydel and Tackenberg 2016 for a discussion in crops). Other studies have identified differential effectiveness of seed dispersal by animal vectors in different conditions (Martorell and López 2014). As an example, somatic seed polymorphisms in *Picris echioides* have been found to lead to differential dispersal success by small mammals (Sorensen 1978).

### Change in external conditions

It is worth noting that global environmental change could mediate rapid changes in seed dispersal without changes in dispersal traits. In a seed release experiment, Cabra-Rivas *et al.* (2014) found that stream structure could affect the seed dispersal of the hydrochorous, invasive tree *Ailanthus altissima.* In particular, dispersal distances increased in degraded reaches because of a loss of complexity and a lack of potential retention zones. Similarly, Greene and Quesada (2011) showed that dispersal distances in the wind-dispersed weed *Tragopogon dubius* were highly influenced by wind speeds and turbulences. Marchetto *et al.* (2010) show that the structure and height of surrounding vegetation strongly affects dispersal. For zoochorous transport, seed dispersal could be affected simply by the presence and density of dispersers. This is most apparent if the absence of the animal disperser leads to dispersal network disruptions (Rogers *et al.* 2017). In a theoretical study, Jones *et al.* (2017) showed that animal traits such as movement distance, activity level and gut retention time interacted with habitat loss such that seed dispersal distances were largest under intermediate habitat loss. Garcia *et al.* (2016) used an isotope-based technique to track bird-mediated seed dispersal and found that small differences in seed and animal traits, for example phenology patterns, could lead to large differences of seed dispersal in response to landscape structure. Morales *et al.* (2013) developed a multispecies mechanistic model of seed dispersal based on frugivore behavioural responses to landscape heterogeneity. Their results indicated that seed dispersal was strongly affected by the frugivore species composition, landscape heterogeneity and the presence of congeneric plant species. If an adult plant can perceive the changed environment, there is potential for plastic responses in fruit or seed traits via epigenetic regulation (Karban and Orrock 2018). If seeds or diaspores with particular traits have higher fitness in these changed abiotic or biotic environments, then there is potential for natural selection and rapid adaptation.

### Change in animal disperser traits

Plant seed dispersal could also change in response to rapid changes in the animal disperser traits. The interaction between changes in plant dispersal and animal dispersal has rarely been studied explicitly. However, density-dependent dispersal in animals has been widely addressed, either alone (Ventura *et al.* 2017; Morton *et al.* 2018) or in interaction with habitat heterogeneity (Zhang *et al.* 2018), personality (Cooper *et al.* 2017) or gender (Paris *et al.* 2016). Many biotic drivers have been studied including the presence of predators (Payo-Payo *et al.* 2018) and parasites (Terui *et al.* 2017), competition (Katz *et al.* 2017), resource availability (Powell and Bentz 2014; Pope and Jha 2017) and quality (Pepi *et al.* 2016; Christy *et al.* 2017; Katz *et al.* 2017), and habitat fragmentation (Moraes *et al.* 2018). Other studies have focused on abiotic drivers affecting animal movement such as ocean acidification (Rossi *et al.* 2018), and climatic conditions including drought (Rozen-Rechels *et al.* 2018), winter weather conditions (Gurarie *et al.* 2017) and wind during migration (Vansteelant *et al.* 2017). Other factors contributing to variation in animal movement include ontogenetic shifts in dispersal (Dahirel *et al.* 2017; Bombieri *et al.* 2018) and individual personality (Cote *et al.* 2013; Michelangeli *et al.* 2017) though the lability of personality is uncertain (Ochocki and Miller 2017; Szűcs *et al.* 2017).

From an evolutionary perspective, changes in animal dispersal have been addressed in the context of delayed dispersal in the evolution of cooperative breeding (Wild and Korb 2017), evolution of density-dependent dispersal with range expansion (Fronhofer *et al.* 2017), the single gene pleiotropic effect on local dispersal (Edelsparre *et al.* 2014), morphological changes and specialization for limited resources (e.g. Darwin’s Finches) (Grant and Grant 2006) and the quantitative genetic variation underlying local dispersal and the rate of exploration of novel environments (Korsten *et al.* 2013). However, it is unclear how rapidly these changes occur, but experimental studies in guppies have shown rapid evolution (in several traits including escape) in only a few generations as a response to predator presence (Reznick *et al.* 2008). In fact, much of our current understanding of rapid evolution comes from animal studies (Hairston *et al.* 2005; Ellner 2013) including a couple of replicated experiments in natural systems (Losos *et al.* 1997; Reznick *et al.* 1997) demonstrating its potential.

## Potential Mechanisms Driving Rapid Response of Dispersal Traits

Having reviewed the evidence for rapid changes in seed dispersal traits in response to changing environments, a second question is how this occurs. Research on the mechanisms for rapid change in seed dispersal traits first aims to identify if the trait change is due to plasticity or genotypic change. For plastic traits, the next steps are to understand the mechanism leading to trait change and determine if the change is heritable. For genetic trait changes, the next step is to identify the region under selection and the strength of selection. Studies approach these topics from different directions in observations, experiments and models, some of which we highlight in this section. Though the exact mechanisms contributing to variability and rapid changes may result in similar outcomes for ecology, at least in the first instance, the issue of heritability is important, and the long-term effects of evolutionary and plastic responses may differ. For example, natural selection on standing genetic variation may evolve rapidly in response to environmental changes (Ellner 2013), and is a widely accepted mode of rapid evolution. Epigenetic research is starting to provide a mechanistic bridge between genetic and plastic variation (Moler *et al.* 2018) and can also occur rapidly. Whether epigenetic inheritance can contribute to rapid adaptive evolution remains a highly contested topic in evolutionary ecology and we hence discuss the potential/debated importance of phenotypic plasticity, the role of epigenetics and how these two processes are involved in non-heritable rapid changes as well as heritable adaptive evolution with implications for seed dispersal.

### Phenotypic plasticity

Phenotypic plasticity is classically defined as the range of phenotypes that a single genotype can express as a function of its environment (Nicotra *et al.* 2010). Years of common garden studies and norms of reaction analyses have shown that plants have a wide range of phenotypes under different environmental conditions (Pigliucci 2005; Futuyma 2017). A variety of manipulations of environmental conditions and selections of plant individuals from different provenances or genotypes have been combined in common garden experiments or in *in vivo* settings to measure their effects on different plant traits and distinguish between plastic and genetic responses. Effects of climate changes on dispersal are either simulated with different temperature, moisture or precipitation treatments (see, for example Teller *et al.* 2014, 2016) or tested along environmental (Martín-Forés *et al.* 2017) or geographic gradients (LaRue *et al.* 2018). They are also investigated in transplant experiments with plant individuals from across their ranges or from different provenances (Leiblein-Wild and Tackenberg 2014). In the field of invasion biology, rapid responses to environmental changes are primarily addressed to identify invasion success of exotic species (Davidson *et al.* 2011; Matesanz *et al.* 2012; Huang *et al.* 2015). Here, studies focus on rapid changes in dispersal traits to explain invasion success by either assessing trait responses in the native vs. non-native range (Martín-Forés *et al.* 2017); at the edges of non-native ranges (Pichancourt and van Klinken 2012) or through the comparison of invasive and closely related, non-invasive species (Sendek *et al.* 2015; Doudová *et al.* 2017).

Empirical studies of rapid changes in plant traits are now increasingly used in meta-analyses to generalize responses and identify traits that are most affected (i.e. Davidson *et al.* 2011; Cochrane *et al.* 2015). While empirical studies (specifically experimental manipulations) convincingly show plasticity in phenotypic responses to changes in environmental conditions, the mechanisms behind the responses remained elusive for a long time. In recent years, trait measurements are increasingly combined with different molecular methods and hence allow a more detailed interpretation of observed variation (e.g. Molina-Montenegro *et al.* 2018).

### Epigenetics

Though the definition of epigenetics is hotly debated at the moment (Greally 2018), we use the definition of Moler *et al.* (2018) where epigenetics is defined as the study of changes in gene expression that are not strictly due to changes in the underlying DNA sequence; some of the changes may be heritable. At the molecular level, epigenetics is a regulatory mechanism of phenotypic plasticity controlled by environmental cues resulting in differential gene expression. Not only is epigenetics the explanation for the unexplainable, but it may be the main contributor to phenotypic plasticity (Richards *et al.* 2017; Moler *et al.* 2018). There are several mechanisms of epigenetic regulation that may be important to seed dispersal, including chemically controlled DNA histone modification and transcriptional and translational interference via non-coding RNAs (e.g. siRNAs) (Moler *et al.* 2018), but the majority of epigenetic regulation studies of plant traits tend to focus on the role of DNA methylation of cytosine (5mC) (Birney *et al.* 2016; Quadrana and Colot 2016; Richards *et al.* 2017; and reviewed in Moler *et al.* 2018). This is in part because CG, CHG and CHH (where H can be A, C or T) nucleotide methylation patterns can affect the silencing of genes, including transposable elements, and ultimately gene expression of ecologically important traits (Law and Jacobsen 2010) and because epigenetic controls such as histone modification are not thought to be strictly heritable (but see Nightingale *et al.* 2006) from one generation to the next (Eichten *et al.* 2014). In plants, there is an increasing body of evidence illustrating that epigenetic variation (5mC methylation) is widespread in natural populations (Niederhuth *et al.* 2016). The take-home message is that the different forms of epigenetic modifications affect gene expression by differentially providing access to the underlying genetic code (Richards 2006).

Heritable or transmissible (intergenerational (F0 to F1), multigenerational (F1 to F2) and transgenerational (>F2)) epigenetic marks (e.g. 5mC) are often associated with stable alterations in gene expression associated with environmental cues (Heard and Martienssen 2014; Taudt *et al.* 2016). Most epigenetic studies in plants are associated with intergenerational inheritance of methylation patterns during cell proliferation (Quadrana and Colot 2016). For example leaf shape (prickly vs. non-prickly) is associated with DNA methylation profile in *Ilex aquifolium* (Herrera and Bazaga 2013). Epigenetic regulation of plant height has been documented in association with drought and temperature (Mirouze and Paszkowski 2011; Kim *et al.* 2015). Variation in seed size was found to be associated with 5mC in *Lavandula latifolia* (Alonso *et al.* 2018). Additionally, bud-burst timing associated with temperature during embryogenesis contributes to seed production in *Picea abies* (Yakovlev *et al.* 2014, 2016) and has been shown to be plastic and under epigenetic control. Recent work has highlighted the role of epigenetic regulation of fruit ripening illustrating the potential for rapid changes in fruit traits (Giovannoni *et al.* 2017). Epigenetic controlled plasticity may be an important adaptive response when shifts in allele frequency are slow relative to the pace of global change, thus providing an alternative rapid response strategy (Braeutigam *et al.* 2013).

**Figure 2.**
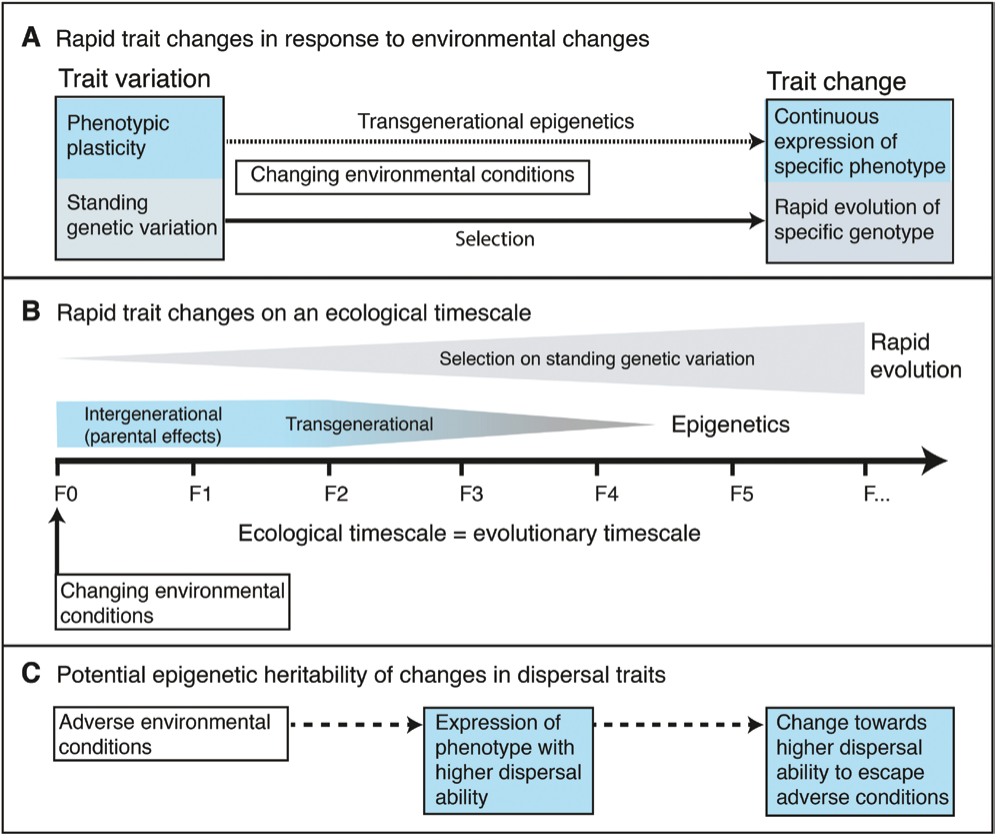
Rapid trait changes in response to environmental change (A) in relation to the eco-evolutionary timescale (B), exemplified for potential epigenetic heritability of changes in dispersal traits (C). (A) Variation of traits in a population can be caused by phenotypic plasticity of one genotype or the standing genetic variation (several genotypes). Changes in environmental conditions could trigger epigenetic responses, with the expression of a specific phenotype. Environmental changes could also select for a specific phenotype based on a specific genotype. If epigenetic effects are transgenerational, they might lead to the continuous expression of a specific phenotype, potentially affecting rapid evolution. Continuous selection for a specific trait from the standing genetic variation can lead to rapid evolution in response to changing environmental conditions. (B) For rapid trait changes, the evolutionary timescale matches the ecological timescale, i.e. trait changes happen in direct response to environmental changes. Epigenetic parental effects might affect only one or two generations, but transgenerational epigenetic effects can span multiple generations. Rapid evolution can occur over a low number of generations, for example by selection on the standing genetic variation. (C) Epigenetic effects in response to environmental changes might be heritable. In this example, epigenetics lead to the expression of a phenotype with higher dispersal ability in response to adverse environmental conditions to escape these conditions. If the epigenetic effect is heritable, the population might change towards having higher dispersal ability and hence be able to escape adverse environmental conditions better.

Transmission of epialleles from one generation to the next is far less certain (Quadrana and Colot 2016), and we still do not know how common it is in nature. However, multigenerational (Schmitz *et al.* 2011), and transgenerational epigenetic inheritance (Richards 2006; Hagmann *et al.* 2015) has been documented in some model plant systems and could provide a framework for testing the transmission of phenotypic plasticity (Herman *et al.* 2014; Whipple and Holeski 2016). Epigenetic regulation is a mechanism that enables changes in the environment to switch gene expression on or off, providing a potential means of rapidly altering traits important for dispersal (within one generation) and if stable and heritable may contribute to adaptive evolution (Fig. 2).

### Rapid evolution

In many organisms, evolution can occur on timescales equivalent to a few generations leading to rapid adaptation to changing environments (Ellner 2013) (Fig. 2B). Rapid adaptive evolution may be the product of selection on standing genetic diversity, or a product of other heritable variability such as epigenetically regulated phenotypic plasticity. Recent advances have been made in the study of invasive plants to identify mechanisms that facilitate their rapid spread using different genotypes or transplants from populations along expansion ranges in common garden experimental settings (Huang *et al.* 2015; Williams *et al.* 2016). For example, Huang *et al.* (2015) tested whether changes in the plume loading of the invasive *Mikania micrantha* in China was a result of rapid evolution due to selection or phenotypic plasticity by measuring seed traits along the range expansion distance. Seeds along this expansion range were also grown in common garden experiments and measurements of their F1 generation were compared to the field measurements. The comparison showed that dispersal traits in the common garden experiment were not significantly related to the expansion distances. However, the dispersal ability at the edge of the range was higher than in populations towards the source of the spread, indicating rapid and adaptive evolution through selection for higher dispersal ability rather than the plasticity of the dispersal ability (in this case plume load). Additionally, the role of natural selection and rapid evolution (due to spatial sorting of favourable dispersal-related alleles) may be an important factor in sweeping beneficial seed dispersal traits to high frequencies within expanding populations (Ochocki and Miller 2017).

Studies of rapid evolution of dispersal traits focus almost exclusively on genetic components, be it the observations or measurements of heritable traits in experiments (e.g. Cheptou *et al.* 2008) or measurements of traits combined with genetic or genomic approaches (Zenni *et al.* 2014; Williams *et al.* 2016). For example, Cheptou *et al.* (2008) sampled dispersing and non-dispersing seeds of *Crepis sancta* in highly fragmented, small habitat patches in an urban area. Observing that the proportion of non-dispersing seeds was much higher in fragmented small patches compared to unfragmented populations and knowing that the ratio of non-dispersing to dispersing seeds is heritable, they concluded that this pattern is evidence for rapid evolution over a few generations due to higher costs of dispersal in fragmented urban populations. Costs of evolution of seed dispersal traits in fragmented landscapes could lead to fitness advantages, but also evolutionary suicide in cases where reduced dispersal evolves and eventually leads to small isolated populations and the accumulation of deleterious alleles (Bonte *et al.* 2012; Corlett and Westcott 2013).

We note that our current understanding of the role of epigenetics and phenotypic plasticity of seed dispersal is progressing, and more work is needed. One way that these mechanisms could be combined to contribute to rapid changes in seed dispersal is if epigenetics (whether intergenerational or transgenerational) acts as interpretive machinery of the underlying genetic diversity and thus regulates phenotypic plasticity. As phenotypic plasticity can create diversity in phenotypes increasing the options for selection to act upon, it is likely in some cases to be adaptive when the expressed phenotype provides the highest level of fitness. Then natural selection on the adaptive phenotypes leads to evolution of these genes. This means that phenotypic plasticity and epigenetics may provide a means for rapid evolution of seed dispersal traits. We tend to agree with Price *et al.* (2003) and Haig (2007) and argue that epigenetic and genetic components probably both contribute to population differentiation and influence adaptive genetic evolution on ecological timescales. In sum, epigenetic mechanisms regulating phenotypic plasticity could contribute to rapid evolution of seed dispersal.

## What Is the Consequence of Rapid Change in Seed Dispersal for Population Persistence and Spread?

A final question is how important the reviewed changes in dispersal parameters are for population persistence and spread under global change (Snell et al. 2019). The question is challenging to answer, because few studies have explicitly linked seed dispersal to successful establishment of adult plants, and thus demonstrated demographic consequences to changes in dispersal (Traveset *et al.* 2014), despite the widespread acceptance of dispersal as an important factor in population dynamics and spread. One notable exception is the example of the wind-dispersed thistle. Skarpaas and Shea (2007) estimated the spread rate of two invasive thistles (*Carduus nutans* and *C. acanthoides*) in North America, and showed that plant traits such as seed release height and seed weight affect dispersal distances. In subsequent studies, it was demonstrated that warming led to higher seed release probabilities in *C. nutans* (via changes in the plastic traits of seed release height and seed weight, Fig. 2) (Teller *et al.* 2014) as well as higher seed release probability in wind tunnels (Teller *et al.* 2016), effectively increasing the spread rate significantly under warmer climate scenarios. Zhang *et al.* (2012) found parental effects of warming on the early life stages of *C. nutans* might also be heritable. Which mechanisms cause the phenotypic plasticity in those dispersal traits is still unknown.

It is important to note that predictive models rarely take into account variation in dispersal traits (see Box 1 for an example of how this can be achieved) although inclusion of such variation could greatly alter predictions under global change (Travis and Dytham 2002; Clobert *et al.* 2009; Burton *et al.* 2010; Jongejans *et al.* 2011; Travis *et al.* 2013; Bourne *et al.* 2014). There is some evidence that the ability to rapidly change dispersal traits can influence the success of biological invasions and extend the range of populations to include suboptimal environments (Pichancourt and van Klinken 2012). This suggests that the ability to rapidly change dispersal traits may be relevant to the spread rates of species and to how effectively populations can be expected to adapt to climate change. We ran numerical experiments with our model (Box 1) where we considered the effects of plasticity in seed dispersal traits on the rate of invasion of a population into a periodically fragmented one-dimensional environment with alternating regions of more or less favourable habitat. We found that in some cases the model predicted that trait plasticity could lead to increased invasion speed, but that depends on the details of the environment and the specific traits and plastic responses of the population (for details, see the **Supporting Information—File S1**). We expect that more detailed study based on the model will lead to additional insights on when and why plasticity in dispersal traits might be advantageous. In addition to our model, some models addressing invasive species have looked at the evolution of dispersal (Hastings *et al.* 2005; Perkins *et al.* 2013) in the context of spatial spread and these models could provide a starting point for further work. Models that have looked at whether a species can keep up with a moving suitable habitat (e.g. Berestycki *et al.* 2008) have not previously included plastic responses or genetic or epigenetic adaptation (or details of dispersal) but could and should be modified to do so.

**Box 1**

We use an integro-difference equation framework to model total seed dispersal pathways that allows us to examine and compare plasticity strategies (see **Supporting Information** for details of the model). Here the plant produces seeds of various types, where the seed type *i* corresponds to seeds produced by the plant whose dispersal pathway is governed by the composite integro-difference kernel *k*_*i*_. Here the index *i* may be discrete or continuous, and the proportion $\alpha_{i}(x,t)$ of seeds at (x,t) of type *i* is non-negative and satisfies $\sum_{i}\alpha_{i}=1$ in the discrete case and $\overset{}{\int }\alpha_{i}di=1\;$ in the continuous case. Summing over *i* in the discrete case or integrating over *i* in the continuous case and carefully tracking seed production, dispersal and establishment, the model will take the adult plant density P(x,t) at location *x* in spatial region Ω and time *t* to the next generation adult plant density P(x,t+1). The model can be expanded to compare the evolution of different plasticity strategies by dividing the overall population into different subclasses, which produce different proportions of the possible seed types. This is done by indexing the subclasses (e.g. by the index *j*, for j=1,2,…,J with density $P^{\;j}(x,t)$ for the *j*th subclass) letting the proportion $\alpha_{i}$ of seeds of dispersal type *i* vary with subclass, so that in subclass *j*$\alpha_{i}$ is replaced with $\alpha_{i}^{\;j}$. One then elaborates the model into a competition model for $\left(P^{1},P^{2},\ldots ,P^{J}\right)$ in the spirit of adaptive dynamics. In the elaborated model one may track the transient and/or asymptotic distributions of $\left(P^{1},P^{2},\ldots ,P^{J}\right)$.

**Figure 3.**
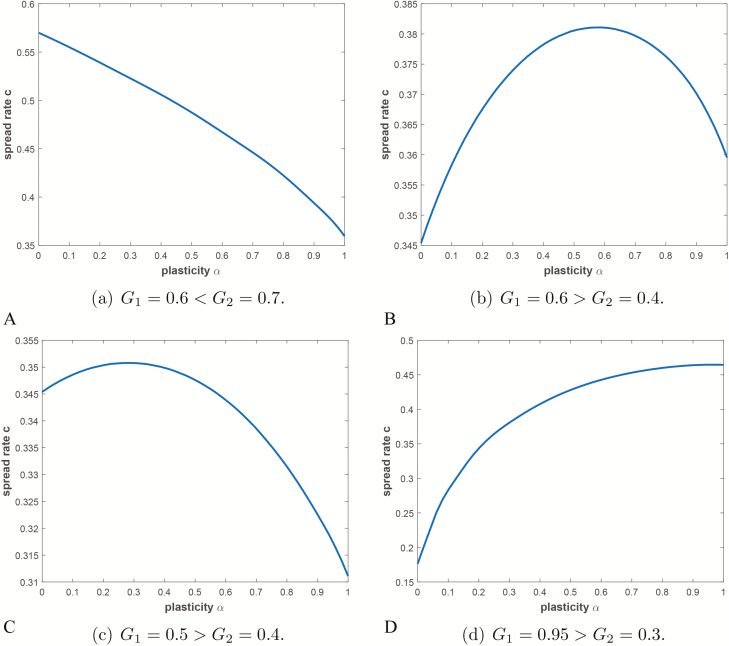
The figure shows the spread rate of the plant species in question as a function of the plasticity parameter *α*. Here *G*_1_ and *G*_2_ represent overall survival rates associated to the two seed dispersal pathways.

We carry out numerical simulations of the model under the simplifying assumption that the plant is an annual that produces two types of seeds with distinct dispersal pathways corresponding to the same underlying probability distribution but with different parameters. We consider both Gaussian and Laplace distributions. In this case $\alpha_{2}=1-\alpha_{1}\;$. We also assume that the landscape is one dimensional and spatially periodic with alternating patches of constant poorer resource availability and constant richer resource availability. Our simulations examine the rate of spread of the population as a function of the degree of plasticity $\alpha =\alpha_{1}$ on [0,1]. We show that a mixed plasticity strategy can sometimes maximize the spread rate depending upon the overall survivability of each seed type. We illustrate this below for the case of Gaussian kernels (Fig. 3). Details of the model and the simulations are given in the **Supporting Information**.

Previous theoretical work acknowledges the existence of both random and non-random reasons for trait variability, but most mathematical or simulation approaches then concentrated on heritable or non-heritable random variability. For example, Albert *et al.* (2011) showed in a simple simulation of wind dispersal that variability in the terminal falling velocity of seeds increases the probability of long-distance dispersal. Thus, dispersal distances would be underestimated when considering only the average falling velocity in the model. This is a particular example of Jensen’s inequality, which states that if the relationship between variable traits and a response is non-linear, then predictions based on the mean trait (rather than the full distribution of trait values) could overestimate or underestimate the response (Bolnick *et al.* 2011; Moran *et al.* 2016). Such non-linear averaging effects induced by random trait variability, as well as the tendency of trait variability to spread risks (portfolio effect), are relatively well understood.

Comparatively less attention has focused on theoretical models to understand the effect of non-random non-heritable or heritable (via hard or soft selection, see, e.g. Messer and Petrov 2013) variation. In general, such variation should improve the performance and adaptive potential of plants to environmental variation (see Malanson (2018) for a simulation study of the role of intraspecific variability and climatic volatility). Imbert and Ronce (2001) showed that heteromorphic *Asteraceae* increase the dispersal of their progeny under environmental stress by varying the relative proportion of wind- and animal-dispersed seed morphs. More interestingly, the plastic increase in dispersal could only be observed under nutrient depletion as a typically longer-lasting environmental stress but not for herbivore pressure as a more unpredictable stress, which corroborates theoretical expectations.

Predicting potential effects of variability in seed dispersal traits on community dynamics is even more complicated than predicting population effects. Despite its complexity, biotic and abiotic interactions and their evolutionary dynamics must be considered to avoid incomplete or erroneous conclusions regarding the mechanisms influencing patterns of seed dispersal (Hand *et al.* 2015; Luikart *et al.* 2018). Concurrently, the effect of phenotypic plasticity on coexistence of competing plants remains largely unclear (Turcotte and Levine 2016). Hart *et al.* (2016) used a theoretical model to test the effects of random variation in demographic rates and competitive parameters (through non-linear averaging) as well as demographic stochasticity. In contrast to common expectations, they found that intraspecific variation could destabilize rather than stabilize coexistence. However, their model concentrated only on random variation and ignored spatial dynamics while dispersal plays a crucial role in metacommunity dynamics and can affect local and regional diversity in a number of ways (Leibold *et al.* 2004). The competition–colonization trade-off predicts that high colonization ability in competitively inferior species could promote coexistence with dominant competitors that are poorer dispersers. The mass effect perspective predicts that species could be rescued from local competitive exclusion by immigration from other source communities. From these theoretical expectations, we could conceive of different ways in which variability in seed dispersal could promote coexistence. For example, plastic increases in dispersal could not only help escape from high intraspecific competition, but also escape from stressful environments. If plant species differ in their adaptive potential, species with a lower adaptive potential could be at a relative disadvantage.

As a complicating factor, seed dispersal in many species is also affected by mutualistic interactions between plant species and their animal seed dispersers, and these interactions can help to stabilize or destabilize coexistence (Jeltsch *et al.* 2013). Differences in the adaptive potential of plants and animal dispersers (e.g. phenological response) may lead to disruption of the seed disperser network or establishment of novel seed dispersal interactions with unclear effect on population and community dynamics (Warren *et al.* 2011; McConkey *et al.* 2012). In the future, more concerted effort is needed to link empirical and theoretical approaches as well as targeted experiments to explore the impact of rapid changes in seed dispersal plasticity in complex community dynamics (Miner *et al.* 2005; Turcotte and Levine 2016).

## Summary, Challenges and Future Directions

Seed dispersal is an essential life-history stage for plants, yet our understanding about the details of this process remains limited. This is both because of the high variability in effective dispersal due to exogenous factors (climate, wind, terrain), but also because of the high intrinsic variability of dispersal-related traits of seeds as well as their dispersers, associated with phenotypic variation (Herrera 2017). Moreover, challenges to studying rapid changes in dispersal exist because there are extensive logistical constraints to untangling these processes. In this perspective essay, we have argued that the variability in dispersal-related traits plays an essential role in regulating dispersal processes, in particular in non-equilibrium situations, and thus deserves closer attention by ecologists studying the role of seed dispersal, in particular in connection with the adaptation to global environmental change.

We have shown that there is evidence and potential for rapid regulation of seed dispersal processes via heritable or non-heritable variation, both in plants, and in seed dispersers. The effects of this variation for plant fitness and plant community dynamics are less clear. While the induced variation in plants may be adaptive (i.e. fitness enhancing), it is very difficult to demonstrate this, due to the necessity of understanding the entire life cycle of a plant to determine fitness. The effect of behavioural changes of seed dispersers is even more elusive, in the sense that they will optimize their own survival, an objective that is not necessarily aligned with the fitness of their associated seed plants. Together, these processes engender substantial uncertainty about the stability of seed dispersal processes and thus plant reproduction under different environmental conditions.

We believe that future research directions focused on understanding how rapid modifications in seed dispersal can change the capacity of species to respond to global ecological change can focus on three points.

First, we urgently need improved data on the range of subindividual variation associated with seed dispersal traits. We must dissect the degree to which the subindividual variability is the product of genetic variability or phenotypic plasticity. This can be accomplished using targeted experiments, molecular approaches and common gardens (Whipple and Holeski 2016; Moler *et al.* 2018). Further, to the degree possible, the proportion of phenotypic plasticity that is associated with patterns of DNA methylation (epigenetically induced variation) needs to be quantified. If phenotypic plasticity is determined to be under epigenetic control (e.g. 5mC) then the heritability needs to be quantified, this can be done using multigenerational reciprocal common garden experiments. If variation in seed dispersal traits (e.g. seed size or mass) are unexplained by genotype or environment variability, and persist for multiple generations, then this would suggest that epigenetic variation could be adaptive and heritable. This task is complicated by the number of different traits that contribute to seed dispersal (seed, plant and disperser) and which vary at the subindividual scale. The empirical findings must be deposited in comprehensive databases that contain information on mean seed dispersal trait values and standard deviations; ideally with environmental covariates identified.

Second, theoretical models that describe these changes broadly, based on identified plant traits and the adaptive landscape on which dispersal traits operate for each plant, need to be developed so that we can predict how seed traits may change in response to biotic and abiotic factors and stressors. Our model (Box 1) begins to address this research direction but must be expanded upon to include real-world examples which can inform empirical research and decision-making. Similar models should be constructed for key dispersal functional groups of plants. A general theory of trait change in plants in response to changes in the biotic and abiotic environment could both guide further empirical tests and link to plant demographic models, improving models of spatial spread (e.g. Neubert and Caswell 2000; Jongejans *et al.* 2011) and dynamic vegetation models (e.g. Hartig *et al.* 2012; Snell *et al.* 2014).

Lastly, in these times of global change, it is crucial to understand which plant species are under threat because they are unable to keep up with their environmental niche. Including rapid responses in seed dispersal traits will allow us to refine our predictions for various species and inform our decisions about how to respond proactively to global change to minimize population/species loss in the future. If we combine our empirical understanding of heritable and non-heritable mechanisms of seed dispersal with theoretical and mathematical modelling of dispersal pathways, we are likely to improve predictions and improve our ability to predict further into the future for best- and worst-case scenarios.

## Sources of Funding

The ideas and discussion for this manuscript stemmed from the authors participation in a National Science Foundation-funded (NSF DEB # 1548194) workshop on seed dispersal held at the National Socio-Environmental Synthesis Center (SESYNC, funded by NSF DBI-1052875) in May 2016. RSC, CC, and XY were supported in part by NSF (DMS # 1514752). DZ received funding from the Swiss National Science Foundation (SNF, grant: PZ00P3_168136/1) and from the German Science Foundation (DFG, grant: ZU 361/1-1).

## Contributions by the Authors

J.S.J. and G.P. led the paper, everyone else alphabetical. All authors contributed to discussions, writing and approved the manuscript.

## Conflict of Interest

None declared.

## Supporting Information

The following additional information is available in the online version of this article—

File S1. Model for plants with Plasticity in seed dispersal.

plz020_suppl_Supporting_Information

## Glossary

**Table:**

Term	Definition	Citation
*Adaptive trait*	A trait that has higher fitness in a specific environment.	Charlesworth *et al.* (2017)
*Adaptive and neutral evolution*	Neutral evolution is when alleles and their associated phenotypes change without being selected for (neutral drift), while adaptive evolution refers to changes in allele frequencies due to selection.	(Luikart *et al.* 2018)
*Adaptive phenotypic plasticity*	The part of phenotypic plasticity that improves the fitness of an organism (e.g. adaptation to a spatially or temporally variable environment), and should thus be selected for.	*Sensu* Dudley and Schmitt (1996)
*Dispersal*	The movement of a propagule from its natal source with consequences for gene flow through space.	Ronce (2007)
*Epialleles*	Loci differing in chromatin state among cells or organisms.	Moler *et al.* (2018)
*Epigenetics*	The study of changes in gene expression that are not due to changes in the underlying DNA sequence regardless of heritability.	Moler *et al.* (2018)
*Epigenetic inheritance*	The phenomenon that epigenetic regulation of gene expressions can be maintained during cell proliferation (mitosis) or between generations (meiosis). Intergenerational F0, multigenerational F0–F1 or transgenerational >F2	Quadrana and Colot (2016)
*Local adaptation*	As a result of genotype by environment interactions, and in the absence of other forces and constraints, divergent selection should cause local populations to evolve traits that provide an advantage under its local environmental conditions.	Kawecki and Ebert (2004)
*Reaction Norm*	The range of phenotypes expressed by a given genotype across some environmental gradient.	Via and Lande (1985)
*Parental effects*	An effect of the parental phenotype on offspring phenotype. Epigenetic inheritance from F0 to F1.	Uller (2008)
*Phenotypic plasticity*	The ability of a single genotype to produce different phenotypes when exposed to different environmental conditions.	Pigliucci (2005)
*Rapid evolution*	A genetic change occurring rapidly enough to have a measurable impact within a few generations.	Hairston *et al.* (2005)
*Standing genetic variation*	The genetic variability in a population.	Barrett and Schluter (2008)
