# Supplementary material for: Rapid changes in seed dispersal traits may modify plant responses to global change
Source: AoB Plants. 2019 Mar 28;11(3):plz020. doi: 10.1093/aobpla/plz020 (PMC6548345; doi:10.1093/aobpla/plz020)
Supplement: plz020_suppl_Supporting_Information [file plz020_suppl_supporting_information.pdf]

Model for plants with Plasticity in seed dispersal

The model

Concept: To construct integro-difference models with multiple kernels, allowing for production of seed types corresponding to those kernels in different proportions, depending on conditions, to describe plasticity. We start by formulating a model for a single population with **two seed types** and then comment on how to elaborate and expand it.

The model starts with seed production, then dispersal, then establishment. We will show various rates and parameters as depending on the time and location, but in principle, the dependence on time and space could arise from dependence on population density, local environmental factors which themselves appear as parameters in the model, and so forth.

We use x and y to denote spatial locations. These could be vectors but we do not denote them as such. We use continuous space but discrete space would be similar. Plants produce seeds, seeds disperse, and then seedlings compete for space. We initially consider a single plasticity regime. Table S1 delineates the parameters and variables in the model.

STEP ONE (Seed production): Seeds of type i per area (density of seed production, in effect) produced are given by:

$$\alpha_i(x,t)\left(\frac{b(x,t)}{\gamma_i}\right)P(x,t) \tag{1}$$

Remark 1. In (1), i could be discrete or continuous. In the simplest (discrete) case $i = 1,2,...$, but there could be several types or even a continuous distribution of types.

Table S1: Biological interpretations of parameters and variables.

$P(x,t)$	adult plant density at location x and time t.
$b(x,t)$	level of energy (resources) available for making seeds (per captia) at (x,t).
γ_i	cost of producing seeds of type i, $i = 1,2$.
$\alpha_i(x,t)$	fraction of seeds that are type i at (x,t), $i = 1,2$.
$S_i(x,t)$	density of seeds arriving at x, of type i, at time t.
$k_i(x,y)$	dispersal kernel for seeds of type i.
Ω	spatial region.

Remark 2. In Table S1, $b(x,t)$ could depend on $P(x,t)$. For $\alpha_i(x,t)$, the simple case of two seed types has $\alpha_1 = \alpha$ and $\alpha_2 = 1-\alpha$, $0 \le \alpha \le 1$. Again more complicated situations are possible, i.e., $\alpha_i(x,t)$ might depend on $b(x,t)$ and/or $P(x,t)$. Such dependence would be where we expect plasticity to come into play. In general, for multiple type seeds ($i \ge 2$), $\alpha_i(x,t)$ would be analogous to a probability distribution in that $\alpha_i \ge 0$, $\sum_i \alpha_i = 1$ or ($\int \alpha_i di = 1$). $k_i(x,y)$ could be taken, for example, from the phenomenological or mechanistic kernels in Levin et al., Annu. Rev. Ecol. Evol. Syst. (2003) v.34, pps. 575–604 or kernels developed since then, and these may also depend on t via densities of seed dispersing animals in year t.

STEP TWO (Dispersal):

$$S_i(x,t+1) = \int_\Omega k_i(x,y)\alpha_i(y,t)\left(\frac{b(y,t)}{\gamma_i}\right)P(y,t)dy \tag{2}$$

Dispersal is modeled by a spatial integral of the product of the produced seeds of type i from Step one and the dispersal kernel $k_i(x,y)$, the probability of an individual moving from location y to x. In other words, the seed density of type i in the next generation, $S_i(x,t+1)$ arises by tallying arrivals at x from all possible locations y.

STEP THREE (Establishment):

This could be done in various ways. Here we have thought about it in terms of a "metapopulation" type of competition for space, but in continuous space. (The key idea here is that population growth comes from seeds colonizing empty space, and thus is tightly linked to dispersal. In this way, the model is more akin to metapopulation models than reaction-diffusion models where dispersal and growth are independent processes.) Parameters and variables included in Step Three appear in Table S2.

Table S2: Biological interpretations of parameters and variables.

$K(x)$	density of suitable sites at location x.
$\sigma(x,t)$	fraction of adults surviving from time t to $t+1$, at location x.
$g_i(x,t)$	probability of germination and growth of seed type i.
$S(x,t)$	density of germinated seeds at location x, at time t.
$F(S)$	fraction of available space that will be colonized by a rain of seeds at level S.

Remark 3. In Table S2, K could depend on t if there is environmental change. g_i will be more

important if we have competing populations. In the single population case it measures something like the per-capita contributions of seed types arriving at point x relative to each other.

If $\sigma(x,t)$ is defined as in Table S2, then at time $t+1$ the fraction of the total number of sites at location x that is occupied by adults would be $\frac{\sigma(x,t)P(x,t)}{K(x)}$, so the fraction of sites available would be $1-\frac{\sigma(x,t)P(x,t)}{K(x)}$. These would be colonized by the incoming seeds.

Let $S(x,t+1)=\sum_i g_i(x,t+1)S_i(x,t+1)$ and $F(S)$ be as given in Table S2. We would want $0=F(0)\leq F(S)\leq 1$, with a general assumption that F increases in S. We take a Monod/Holling II form for analytic simplicity:

$$F(S)=\frac{S}{a+S}.$$

A Holling I form

$$F(S)=\begin{cases} F_0 S, & S\leq \frac{1}{F_0} \\ 1, & S\geq \frac{1}{F_0} \end{cases} \tag{3}$$

might also be reasonable in some situations. Note that we can describe S as

$$\begin{aligned} S(x,t+1) &= \sum_i g_i(x,t+1)\int_{\Omega} k_i(x,y)\alpha_i(y,t)\left(\frac{b(y,t)}{\gamma_i}\right)P(y,t)dy && (4)\\ &= \int_{\Omega}\tilde{k}(x,y,t)P(y,t)dy && (5) \end{aligned}$$

where $\tilde{k}$ is a complex kernel built from the k_i' s and other parameters. Then

$$P(x,t+1)=K(x)\left[\frac{\sigma(x,t)P(x,t)}{K(x)}+\left(1-\frac{\sigma(x,t)P(x,t)}{K(x)}\right)F\left(\int_{\Omega}\tilde{k}(x,y,t)P(y,t)dy\right)\right]. \tag{6}$$

At the end of Step Three, one has

$$P(x,t+1)=\sigma(x,t)P(x,t)+[K(x)-\sigma(x,t)P(x,t)]F\left(\int_{\Omega}\tilde{k}(x,y,t)P(y,t)dy\right). \tag{7}$$

The case of annuals ($\sigma(x,t)=0$) is probably simpler than other cases in some ways.

To elaborate the model to compare competing plasticity strategies, we employ the superscript j to denote sub-populations of the species using different plasticity strategies. For each sub-

population we calculate up to equation (6) as before, with all parameters and variables indexed by j, arriving at S^j for sub-population $j(j = 1,2,...,J)$. (We might want $b(x,t)$ to depend on $(P^1(x,t),..,P^J(x,t))$). Then, instead of (7), we would get equations

$$P^j(x,t+1) = \sigma^j(x,t)P^j(x,t) + \left[K(x) - \sum_{j=1}^{J} \sigma^j(x,t)P^j(x,t)\right] F^j\left(S^1,..,S^J\right). \tag{8}$$

for $j = 1,2,...,J$. Here we take

$$F^j\left(S^1,..,S^J\right) = \frac{S^j}{a^j + \sum_{l=1}^{J} S^l} \tag{9}$$

or something similar, (with $S^j = S^j(x,t+1)$.)

(Recall from (6) that S^j includes kernels coefficients g_i^j that could describe competitive strength, and coefficients α_i^j that describe plasticity.)

Remark 4. In (6), we could think of $\alpha = \alpha(x,t,i)$ as corresponding to a continuous probablility distribution in i for each (x,t), and replace $\sum_i$ with $\int_{I_1}^{I_2}(\quad)di$.

Conclusion: Employing a model such as (8), (9) with two populations $(J = 1,2)$ allows us to use pairwise invasibility analysis to study the evolutionary stability of plasticity patterns in dispersal, or related questions.

Numerical results

To illustrate the effects of the plasticity α on the rates of spread and persistence in a spatial heterogeneous environment, we simplify the spatial heterogeneity by considering two types of periodically fragmented patches (Shigesada et al., 1986), denoted as 'good' and 'bad' according to $m(x) := b(x)K(x)$. Next, we denote by l_1 the length of a bad patch and by l the period of the landscape, so that $l - l_1$ is the length of a good patch. Let $F(s) = \dfrac{s}{s+1}$, $\sigma = 0$, $g_i(x) \equiv g_i$, $p(x,t) = \frac{P(x,t)}{K(x)}$ and $G_i := \dfrac{g_i}{\gamma_i}$, which measures the overall survival ability for type i ($i = 1,2$), $\alpha_1 = \alpha$ and $\alpha_2 = 1-\alpha$. Then the model (7) becomes

$$p(x,t+1) = F\left(\int_{\mathbb{R}} \tilde{k}(x,y)p(y,t)dy\right), \tag{10}$$

where $\tilde{k}(x,y) = \sum_{i=1}^{2} \alpha_i G_i k_i(x-y) m(y)$, $\alpha_1 + \alpha_2 = 1$.

Numerically, we first choose the Gaussian kernels $k_i(x) = \frac{1}{\sqrt{2\pi\delta_i^2}} e^{-\frac{x^2}{2\delta_i^2}}$, and

$$m(x) = \begin{cases} m_1, & -l_1/2 < x < l_1/2, \\ m_2 > m_1 > 0, & l_1/2 < x < l - l_1/2. \end{cases}$$

as an example to explore the following scenario: there are only two types of seeds: type 1 and type 2, where type 1 refers to big seeds and type 2 represents small seeds. More specifically, type 1 (big) seeds are assumed to be fewer, but with strong germination properties, and governed by a short range kernel, while type 2 (small) seeds are assumed to be more abundant, but with poorer germination, and subject to a wide ranging kernel. Therefore, this implies $\gamma_1 > \gamma_2$, $g_1 > g_2$, $\delta_1 < \delta_2$.

How does spread rate depend on α ? For our model, because of the form $F(s)$, the overall dynamical behaviors of our model in a periodic habitat would be analogous to that of the classic Beverton-Holt model, that is, the positive solutions of the model system converge to either zero or a unique positive periodic steady state locally uniformly, in other words, colonizing the local region in a periodic (oscillated) fashion when it could survive. (Fig. S1(a) and S1(b)). Recall that the spread rate is an asymptotic rate (slope) defined as the limit of front location $x(t)$ over t as t goes to infinity (Kawasaki and Shigesada, 2007). Since $G_1 = G_2$, small seeds have a larger spread rate due to the associated wider ranging kernel (see Fig. S1(c)). Fig. S2 shows the spread rates under different G_i $(i = 1,2)$. If $G_1 \leq G_2$, the largest spread rates would be the single type with small seeds (see Fig. S2(a)). But if G_1 is greater than G_2 appropriately, the plasticity $\alpha > 0$ might give a larger spread rate with the mixed types of seeds(see Figs. S2(b) and S2(c)). If G_1 is sufficiently larger than G_2, the largest rates would be the single type with big seeds (see Fig. S2(d)). In any case, we could have $c(\alpha) \geq \alpha c(0) + (1-\alpha) c(1)$. A similar result for system (10) with Laplace kernel $k_i(x) = \frac{1}{2a_i} e^{-\frac{|x|}{a_i}}$ is presented in Fig. S3 and S4.

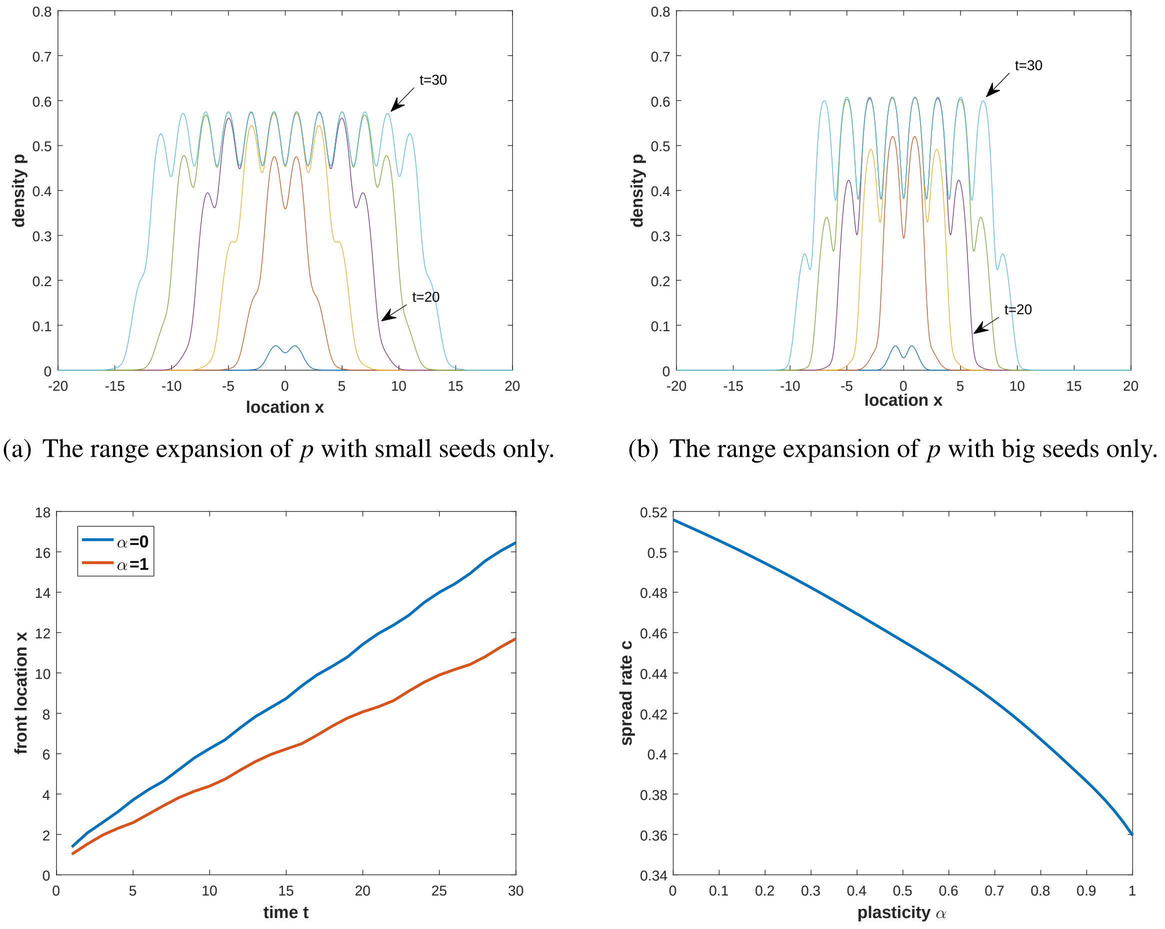

(a) The range expansion of p with small seeds only.

(b) The range expansion of p with big seeds only.

(c) Front location *vs* time.

(d) Spread rate c as a function of α.

Figure S1: We fix $m_1 = 0.2$ and $m_2 = e^{1.5}$, $l = 2$, $l_1 = 0.5$, $\delta_1^2 = 0.1$ and $\delta_2^2 = 0.2$, where the initial population distribution concentrates around $x = 0$. Consider $G_1 = G_2 = 0.6$, iterating for 30 generations. The panels (a) and (b) show the range expansion of the population with a single type of seeds. The panel (c) tracks the front (rightward) location from the simulation and compares it with the associated time when $\alpha = 0$ and $\alpha = 1$, respectively. The panel (d) indicates the spread rate is a decreasing function of plasticity α.

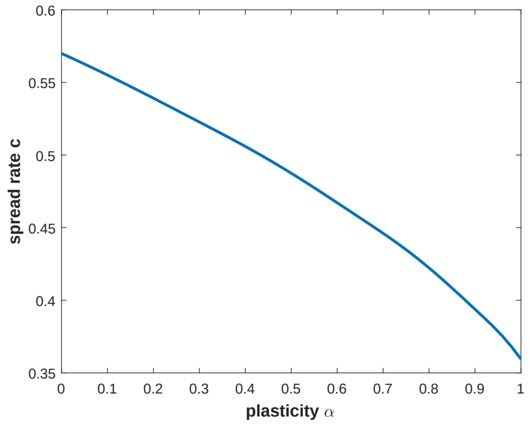

(a) $G_1 = 0.6 < G_2 = 0.7$.

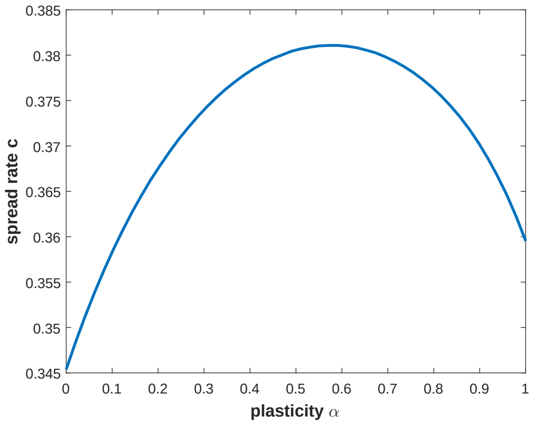

(b) $G_1 = 0.6 > G_2 = 0.4$.

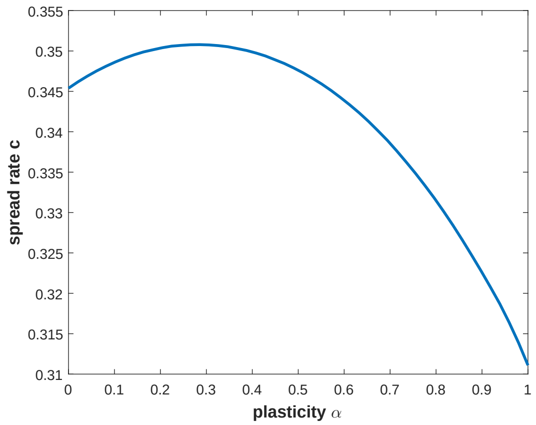

(c) $G_1 = 0.5 > G_2 = 0.4$.

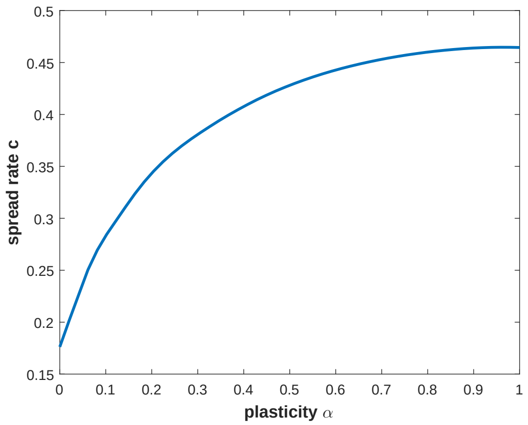

(d) $G_1 = 0.95 > G_2 = 0.3$.

Figure S2: The spread rate c is a function of α with different G_i $(i = 1, 2)$ and the same parameter values as in Fig. S1.

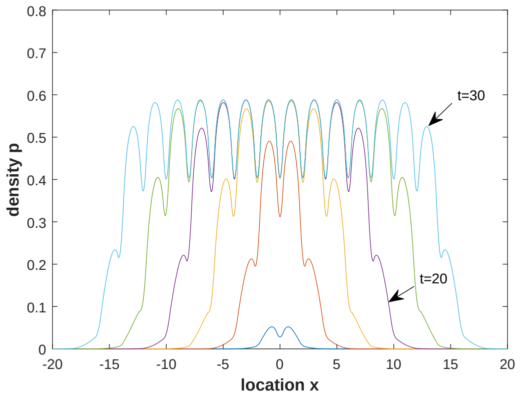

(a) The range expansion of p with small seeds only.

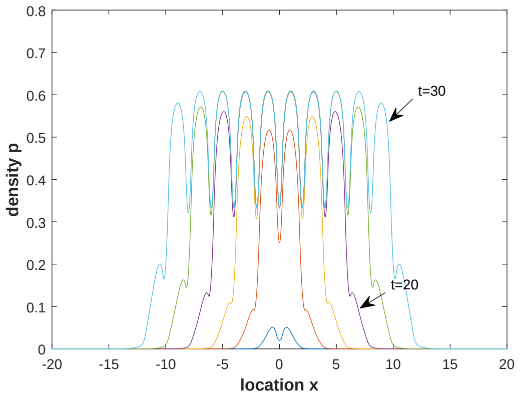

(b) The range expansion of p with big seeds only.

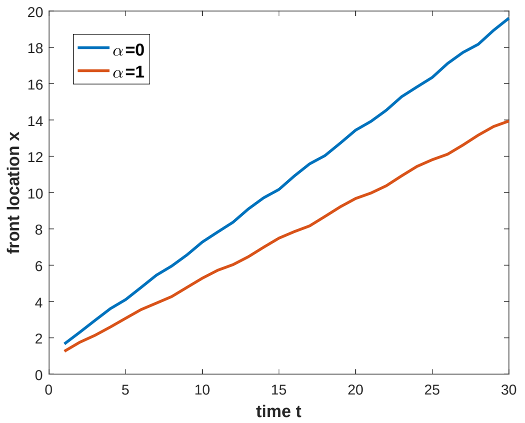

(c) Front location vs time.

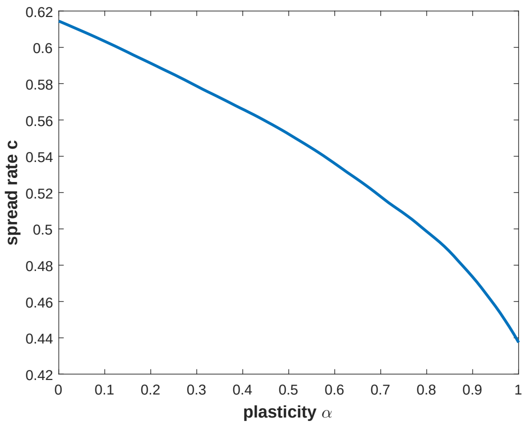

(d) Spread rate c as a function of α.

Figure S3: Propagation dynamics with Laplace kernel. Fix $m_1 = 0.2$ and $m_2 = e^{1.5}$, $l = 2$, $l_1 = 0.5$, $a_1^2 = \frac{\delta_1^2}{2} = 0.05$ and $a_2^2 = \frac{\delta_2^2}{2} = 0.1$, where the initial population distribution concentrates around $x = 0$. Consider $G_1 = G_2 = 0.6$, iterating for 30 generations. The panels (a) and (b) show the range expansion of the population with a single type of seeds. The panel (c) tracks the front (rightward) location from the simulation and compares it with the associated time.The panel (d) indicates the spread rate c is a decreasing function of plasticity α.

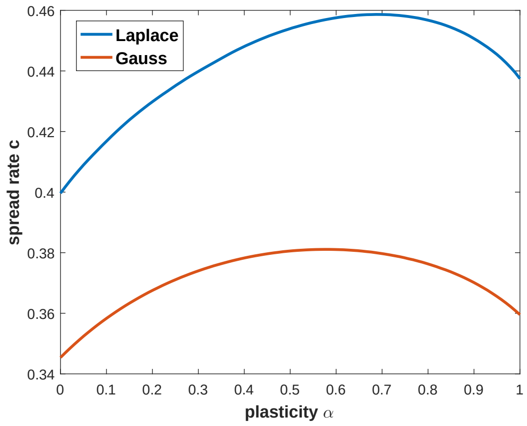

(a) $G_1 = 0.6 > G_2 = 0.4$.

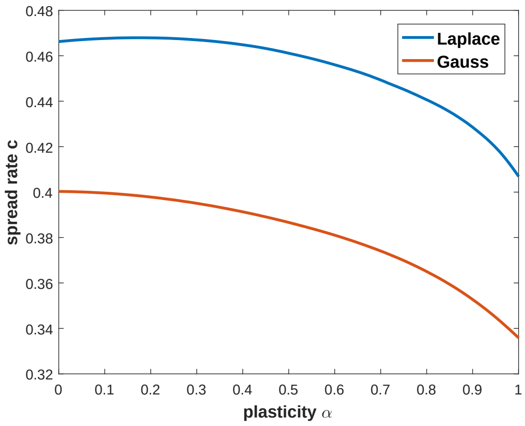

(b) $G_1 = 0.55 > G_2 = 0.45$.

Figure S4: The spread rate c is a function of α with different G_i and the same parameter values as in Figs. S1 and S3 with the same variance.

References

[1] Shigesada N, Kawasaki K, Teramoto E. 1986. Traveling periodic waves in heterogeneous environments. *Theoretical Population Biology*, **30:** 143–160.

[2] Kawasaki K, Shigesada N. 2007. An integrodifference model for biological invasions in a periodically fragmented environment. *Japan Journal of Industrial and Applied Mathematics*, **24:** 3–15.
